# Modulation of cell migration and invasion of thyroid cancer cells using miRNA-enriched extracellular vesicles

**DOI:** 10.3389/fendo.2026.1884738

**Published:** 2026-09-15

**Authors:** Mônica Pezenatto dos Santos, Nathalia Leal Dovaizem, Isabelle Nogueira Silva, Bianca Azevedo de Souza, Letícia Ferreira Alves, Luciana Nogueira de Sousa Andrade, Murilo Vieira Geraldo

**Affiliations:** 1 Department of Structural and Functional Biology, Universidade Estadual de Campinas (UNICAMP), São Paulo, Brazil; 2 Center for Translational Research in Oncology (LIM24), Instituto do Câncer do Estado de São Paulo, Hospital das Clínicas da Faculdade de Medicina da Universidade de São Paulo, Comprehensive Center for Precision Oncology, Universidade de São Paulo, São Paulo, Brazil; 3 Josep Carreras Leukaemia Research Institute, Barcelona, Spain

**Keywords:** cell invasion, cell migration, extracellular vesicles, microRNAs, papillary thyroid carcinoma

## Abstract

**Background:**

Extracellular vesicles (EVs) have been investigated as promising vehicles for delivering drugs and microRNAs (miRNAs) in distinct types of cancer. However, their use as a delivery system in papillary thyroid carcinoma (PTC) remains underexplored. PTC is the most prevalent subtype of thyroid cancer, and despite its good prognosis, a fraction of the cases evolve into aggressive and fatal forms. Here, we investigated whether non-tumor thyroid cells can be engineered to produce EVs-enriched with tumor suppressor miRNAs that could be delivered to PTC cells.

**Methods:**

EVs from NThy-ORI cells transfected with *miR-485-5p* or *miR-495-3p* were isolated by differential ultracentrifugation and characterized by Nanoparticle tracking analysis (NTA), transmission electron microscopy (TEM), and Western Blot. Functional *in vitro* assays were conducted to explore the impact of miRNA-enriched EVs on PTC progression.

**Results:**

Non-tumor thyroid NThy-ORI cells overexpressing *miR-485-5p* or *miR-495-3p* efficiently produced EVs enriched with these miRNAs, which were internalized by PTC cells. Treatment with miRNA-enriched EVs modulated the expression of validated miRNA targets and key epithelial–mesenchymal transition-related genes, leading to a 40% reduction in migration (p<0.0001) and a 60% reduction in invasion in both miRNAs and cell lines tested (p<0.001).

**Conclusions:**

These findings suggest the potential of thyroid-derived EVs as a biologically relevant and promising system for delivering tumor suppressor miRNAs, opening new opportunities to explore this approach as a therapeutic strategy for PTC.

## 1 Introduction

Thyroid cancer (TC) is the most prevalent malignant tumor of the endocrine system, ranking seventh in global incidence in 2024 (1). The most prevalent subtypes, papillary (PTC) and follicular thyroid cancer (FTC), have a good prognosis and high cure rates. However, similarly to other cancers, current therapeutic strategies such as surgery, radioactive iodine therapy, chemotherapy, and targeted therapy still have significant limitations and disadvantages, such as low accuracy and therapeutic efficacy, with recurrent adverse effects (2–4). In this scenario, the use of therapeutic nucleic acids, in particular microRNAs (miRNAs), has been considered a promising strategy to overcome tumor resistance and favor treatment response. One of the most challenging aspects of this therapeutic approach, however, is their efficient and safe delivery to target cells. Recently, the use of extracellular vesicles (EVs) as delivery platforms has gained increasing attention, especially for RNA-based drugs, owing to their ability to protect cargo from degradation and efficiently deliver it to recipient cells. Consequently, the delivery of tumor suppressor miRNAs via EVs to tumor cells has been proposed and could be explored as adjuvant therapy in combination with surgical resection in thyroid cancer (5–7).

Extracellular Vesicles (EVs) have emerged as key mediators of intercellular communication. First discovered 40 years ago, EVs are small particles delimited by a lipid bilayer and released by various cell types. They are classified based on size into large vesicles and small vesicles. Among them, exosomes are often described as small vesicles with a diameter of <200 nm, originating from multivesicular bodies (MVBs); ectosomes, on the other hand, are originated from the budding of the cell membrane and can be classified as small and large EVs (8). These particles naturally contain bioactive molecules, including lipids, proteins, nucleic acids (DNA and RNA), and non-coding RNAs, such as miRNAs, and can efficiently transfer their cargo to recipient cells, allowing them to regulate physiological processes (9).These features, together with their natural ability to target specific cells and tissues, have positioned EVs as highly promising carriers for therapeutic nucleic acids, offering potential advantages over conventional delivery systems, including lipid nanoparticles and viral vectors.

Among the molecules that can be transfer via EVs, miRNAs are a class of small non-coding RNAs, with approximately 22 nucleotides in length, that play a crucial role in gene expression regulation. By pairing with the 3’ untranslated region (UTR) of target mRNAs, miRNAs regulate gene expression at the post-transcriptional level (10). Depending on their target genes, miRNAs can act as either oncomiRs, by inhibiting tumor suppressors, or tumor suppressors, by repressing oncogenes (11).

In recent decades, the use of EVs as nucleic acid delivery systems for cancer therapy, particularly miRNAs, has been explored (7, 9, 12, 13). For example, EV-mediated miRNA delivery was efficient for the delivery of *miR-143* in colon cancer (14)*, miR-145* in lung cancer (15), and *miR-146b* in glioma (16). In PTC, Lee et al. (2015) (17) showed that tumor-derived exosomes overexpressing *miR-146b* and *miR-222* impair the proliferation of recipient cells, highlighting the functional role of exosomal miRNAs in intercellular communication. More recently, Zhang et al. (2025) (18) used EVs derived from hUCMSCs engineered to deliver circRNA to papillary thyroid cancer, promoting tumor suppression via the miR-146b/p53 pathway.

Given the key role of EV miRNA cargo in tumor progression, the use of EVs as a delivery system for tumor-suppressor miRNAs represents a promising therapeutic approach. However, despite these efforts, experimental studies exploring the therapeutic potential of EV-mediated delivery of tumor-suppressor miRNAs in PTC remains poorly explored. Here, we demonstrated that non-tumoral thyroid cells can be engineered to release EVs enriched with tumor-suppressor miRNAs previously characterized by our group, *miR-485-5p* and *miR-495-3p*, and efficiently modulate the progression of PTC recipient cells.

## 2 Materials and methods

### 2.1 Cell culture

Human normal thyroid follicular cells (NThy-ORI) and papillary thyroid cancer cell lines (TPC-1 and BCPAP) were kindly provided by Professor Edna Teruko Kimura (University of São Paulo, Brazil). As a source of EVs, NThy-ORI cells were transfected either with an empty vector (NThy-ORI-ø) or with constructs harboring the genomic region of the miRNAs individually, as previously described (19, 20). Cells overexpressing *miR-485-5p* and *miR-495-3p* were named NThy-485 and NThy-495, respectively, and the EVs derived from these cells were named NThy-485-EVs and NThy-495-EVs.

All cells were cultured in a 37°C incubator with 5% CO2. NThy-ø, NThy-485, and NThy-495 were cultured using RPMI-1640 supplemented with 10% fetal bovine serum (FBS), 2 mM L-glutamine, and 5 μg/mL puromycin. Overexpression of miRNAs was confirmed by RT-qPCR. NThy-ORI, TCP-1, and BCPAP were maintained using RPMI-1640 and DMEM medium supplemented with 5% and 10% FBS, respectively. All culture media included antibiotics (100 U/mL penicillin and 100 μg/mL streptomycin) and 1 μg/mL amphotericin B (ThermoFisher). For EV-related experiments, FBS was previously depleted of vesicles by ultracentrifugation (100.000×g, 16 h, 4 °C). In addition, EVs were treated with RNase A before all functional experiments.

### 2.2 EVs isolation and characterization

EVs were isolated from conditioned medium (CM) employing differential ultracentrifugation as described by Théry et al. (21). NThy-ø, NThy-485, and NThy-495 cells were plated in 150 mm dishes and cultured with RPMI-1640 medium supplemented with EV-depleted FBS for seventy-two hours. Then, CM was collected and submitted to centrifugation at 300×g for 10 min to remove cell debris, followed by 2,000×g for 10 min to remove small debris, apoptotic bodies, and other larger EVs, both at room temperature. Next, the supernatant was ultracentrifuged at 10,000×g for 30 min, followed by 100,000×g for 2 hours at 4 °C (Beckman Coulter Optima XE-90, SW28 rotor, CA, USA). EV pellets were resuspended in phosphate-buffered saline (PBS) and stored at -80 °C for further use.

EVs characterization followed the 2018/2024 consensus guidelines on Minimum Information for Extracellular Vesicle Studies (22). The concentration and size of EVs were measured using Nanoparticle Tracking Analysis (NTA), with 5 measurements of 60s each at 25 °C on the NanoSight NS300 (Malvern, United Kingdom). Transmission Electron Microscopy and Western Blot were employed to analyze the ultrastructure of EVs and classical membrane markers, respectively.

#### 2.2.1 Transmission electron microscopy

The Electron Microscopy Laboratory of the Institute of Biology, University of Campinas, conducted Transmission Electron Microscopy. To confirm EVs’ ultrastructure, four microliters of aliquots of resuspended EVs were applied to 0.5-cm2 glow-discharged Formvar/carbon-coated electron microscopy grids (CF100-cu “carbon film”, Electron Microscopy Sciences - EMS, Hatfield, PA, USA) and incubated at room temperature until the formation of a thin-layer film. Then, the grids were transferred to 2% uranyl acetate for 5 min to contrast the samples. Next, the grids were left to dry and stored in appropriate grid storage boxes. Finally, they were observed under an LEO 906 transmission electron microscope (Carl Zeiss, Germany) at 60 kV.

#### 2.2.2 Western blot

EVs were sonicated with a probe sonicator at a low amplitude using three 5-second pulses. Equal volumes of EVs (25 μL) and 2× Laemmli Sample Buffer (Bio-Rad) were combined and incubated at 95 °C for 5 minutes. Then, EVs were placed into a 10% SDS-PAGE gel (0.375 M Tris, pH 8.8, 0.1% SDS, 10% acrylamide, 0.03% ammonium persulfate [APS], and 0.06% N,N,N′,N′-tetramethylethylenediamine [TEMED]). Proteins were transferred to PVDF membranes using semi-dry transfer and blocked with 5% non-fat milk in 0.1% TBS-Tween for 1 hour at room temperature. Next, the membranes were incubated with primary antibodies overnight at 4 °C, followed by a 1-hour incubation with secondary antibodies at room temperature. Antibodies are shown in **Supplementary Table 1**. Protein bands were visualized using the Western Pico ECL kit (Thermo Fisher Scientific) and captured with the Bio-Rad Chemi-Doc system.

### 2.3 RNA extraction

Total RNA was extracted from cells using TRIzol Reagent (Thermo Fischer Scientific), according to the manufacturer’s instructions. In the final step, total RNA was eluted in 50 μL of RNase-free water for cellular RNA. For EVs RNA isolation, the miRNeasy Tissue/Cells Advanced Mini Kit (QIAGEN, catalog number 217684) was used following the manufacturer’s instructions. Before RNA isolation, EVs were treated with Proteinase K (1mg/mL) for 10 min, followed by RNase A (400 ng/µL; Sigma-Aldrich, MA, USA) for 30 min at 37 °C, as described by Santos, NL et al. (23). RNA concentrations were measured on a Nanodrop ND-1000 (Thermo Fisher Scientific, Waltham, MA, USA).

### 2.4 Expression analysis

For miRNA expression analysis, ten nanograms of total RNA were used to perform cDNA synthesis and quantitative RT-PCR using TaqMan Small RNA Assays (Thermo Fisher Scientific), following the manufacturer’s instructions. RNU6B (Assay ID 001093) and cel-miR-39-3p (Assay ID: 339390) were used as endogenous controls for cells and CM, respectively. Invitrogen’s M-MLV kit (ThermoFisher) and Power SYBR Green PCR Master Mix (Thermo Fischer Scientific, catalog number 4309155) were used for cDNA synthesis and qPCR reactions for the mRNA targets, with 1 μg of total RNA per reaction following the manufacturer’s instructions. RPL19 was used as an endogenous control. Primers are shown in **Supplementary Table 2**. All reactions were carried out on an Applied StepOnePlus (Applied Biosystems/Thermo Fisher Scientific). Data were analyzed using the 2-ΔΔCt method (24).

### 2.5 miRNA uptake by recipient cells

CM was collected from NThy-ø, NThy-485, and NThy-495 cells and cultured for 72 h in 60 mm cell culture plastic dishes. To analyze the incorporation of miRNAs by the tumor cells, TPC-1 and BCPAP cell lines, grown overnight in 12-well plates (10^5^ cells/well) in technical triplicate, were exposed to fresh CM from non-tumor cells. Importantly, the CM from non-tumor cells was used immediately for the treatment of the cancer cell lines. Total cytoplasmic RNA was extracted, and RT-qPCR was employed to evaluate miRNA expression.

### 2.6 Migration and invasion assays

For the scratch assay, one hundred thousand TPC-1 and BCPAP cells were plated in a 12-well plate in technical triplicate, and EVs from NThy-ø, NThy-485, and NThy-495 were added at a concentration of 10^9^ particles/mL. Twenty-four hours later, a wound was made by scratching the plate with a micropipette tip, and the cells were washed with PBS to remove cell debris. Nikon Eclipse E600 microscope (40x magnification) was used to acquire the photomicrographs immediately (0h) and 16 hours after the scratch-making.

For the Transwell-based migration and invasion assays, 8.0 mm pore membrane inserts (Millipore, MA) were employed. Ten thousand cells were resuspended in media without SFB containing EVs at a concentration of 109 particles/mL and plated in the upper compartment of the chamber. DMEM supplemented with FBS-EV-depleted was used to fill the lower compartment. For the cell invasion assay, Extracellular Matrix Gel (ECM Gel from Engelbreth-Holm-Swarm murine sarcoma - liquid, Bioreagent 8.42 mg) was diluted in DMEM and plated on top of the insert. One hour later, the same number of cells with EVs was seeded on top of the ECM coat. For both experiments, after 16 h, the cell media were removed, and the chamber was washed twice with PBS. The cells in the lower compartment were fixed and stained with 0.5% crystal violet. The Nikon Eclipse E600 microscope (100x magnification) was used to capture the images.

Briefly, for both experiments, five predefined microscopic fields were acquired from each well, corresponding to the center and the four corners of the well. The number of migrated/invaded cells in each field was quantified using ImageJ software, and the mean value from the five fields was used for statistical analysis.

### 2.7 Statistical analysis

GraphPad Prism Software v.8.01 was used to perform statistical analysis. The t-test was used to analyze 173 differences between two groups. Two-way ANOVA was used to analyze differences among three or more groups using Dunnett’s post-test. p ≤ 0.05 was considered statistically significant.

## 3 Results

### 3.1 Non-tumor thyroid cells overexpressing miRNAs produce functionally active EVs *in vitro*

Both *miR-485-5p* and *miR-495-3p* are downregulated in PTC cell lines compared to the non-tumoral NThy-ORI cells (**Figure 1A**). We therefore hypothesized that EVs derived from this cell line could serve as a safe delivery platform for these tumor suppressor miRNAs, as they are expected to lack tumor-promoting components such as oncogenic miRNAs and growth factors, cytokines, immunosuppressive molecules, and metabolites (25, 26). To test this hypothesis, NThy-ORI cells were transfected (or not, in the case of NThy-ø) with the genomic regions of *miR-485-5p* or *miR-495-3p* (**Figure 1B**). Importantly, NThy-ø were transfected with the empty vector, serving as the transfection control. Following transfection, EVs were isolated by ultracentrifugation to determine whether the secreted EVs would be enriched with these miRNAs, and indeed, high levels of the given miRNAs were detected in RNase A-treated EVs, indicating that most of the secreted miRNAs were encapsulated within vesicles (**Figure 1C**).

**Figure 1:**
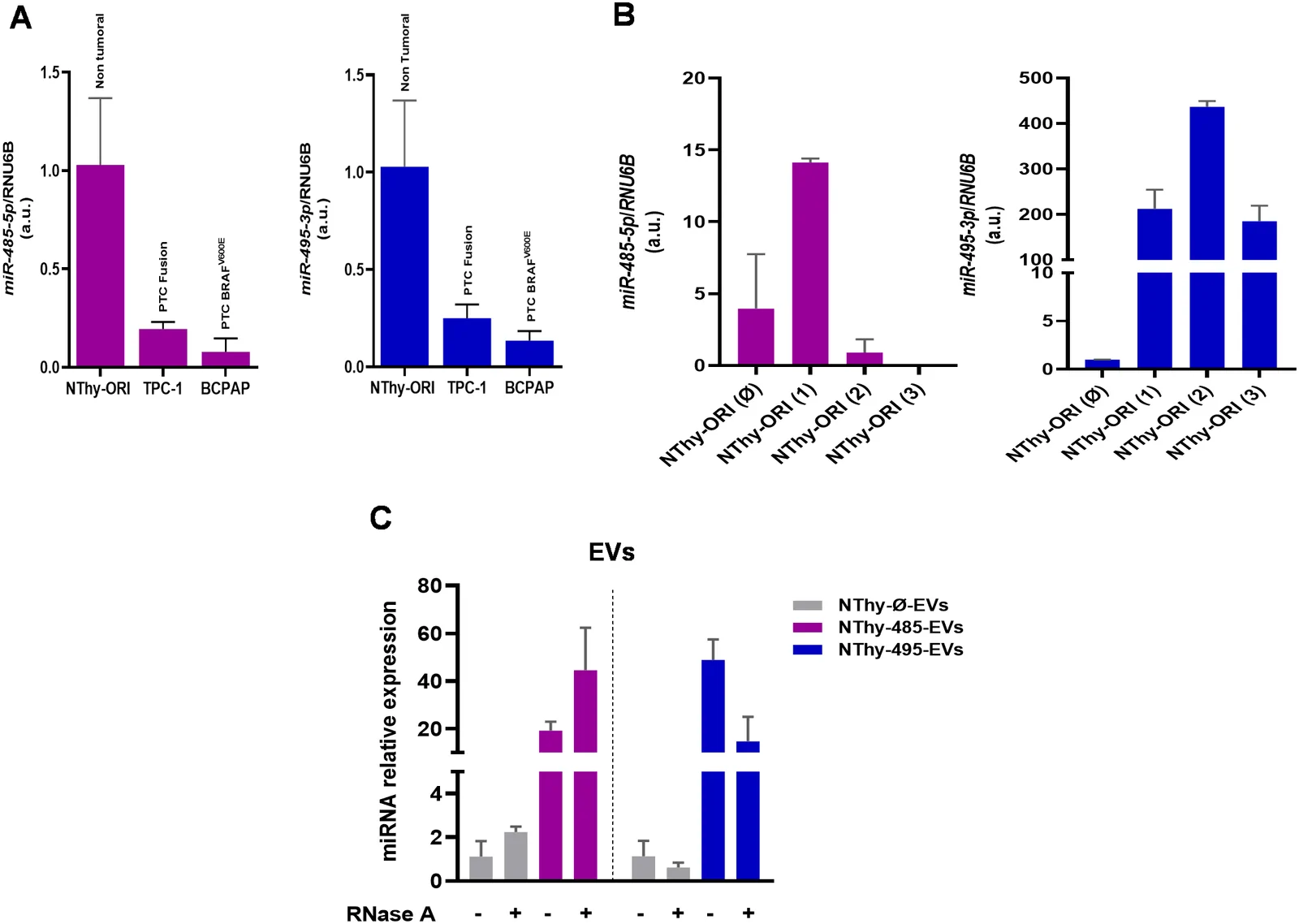
Overexpression of *miR-485-5p* and *miR-495-3p* and secretion of enriched EVs. **(A)** Both *miR-485-5p* and *miR-495-3p* are downregulated in PTC cell lines. **(B)** The bar plot shows the expression of *miR-485-3p* and *miR-495-3p* among the different clones. *RNU6B* was used as an endogenous control. **(C)** Relative expression level of *miR-485-5p* and *miR-495-3p* in EVs derived from transfected NThy-ORI cells. EVs were submitted for treatment with RNase A or not. Data are presented as means ± SD of technical duplicates from three independent experiments (n=3). Statistical analysis was carried out using Student’s t-test.

### 3.2 Isolation and characterization of extracellular vesicles

We next characterized the EVs isolated from non-tumoral NThy-ORI cells (**Figure 2**). EVs concentration and size were characterized using Nanoparticle Tracking Analysis (NTA) on a NanoSight NS300 system (Malvern) (**Figure 2A**). In all three groups, EVs exhibited diameters consistent with small vesicles, smaller than 200 nm (22), with average sizes of 185 nm for NThy-ø, 181 nm for NThy-485, and 183 nm for NThy-495 (data not shown). As expected, TEM also revealed morphology consistent with small EVs in terms of both size and purity (**Figure 2B**). Furthermore, Western blot analysis confirmed the purity of the EV preparations, as demonstrated by the absence of calnexin in the EV pellet, while confirming the presence of classical EV markers, such as CD63 and CD9 (**Figure 2C**). It is important to note that the purpose of this analysis was to verify the presence of EV markers and their purity, rather than to compare markers abundance among EV preparations, as equal volumes, rather than equal amounts of EVs, were loaded for Western blot analysis.

**Figure 2:**
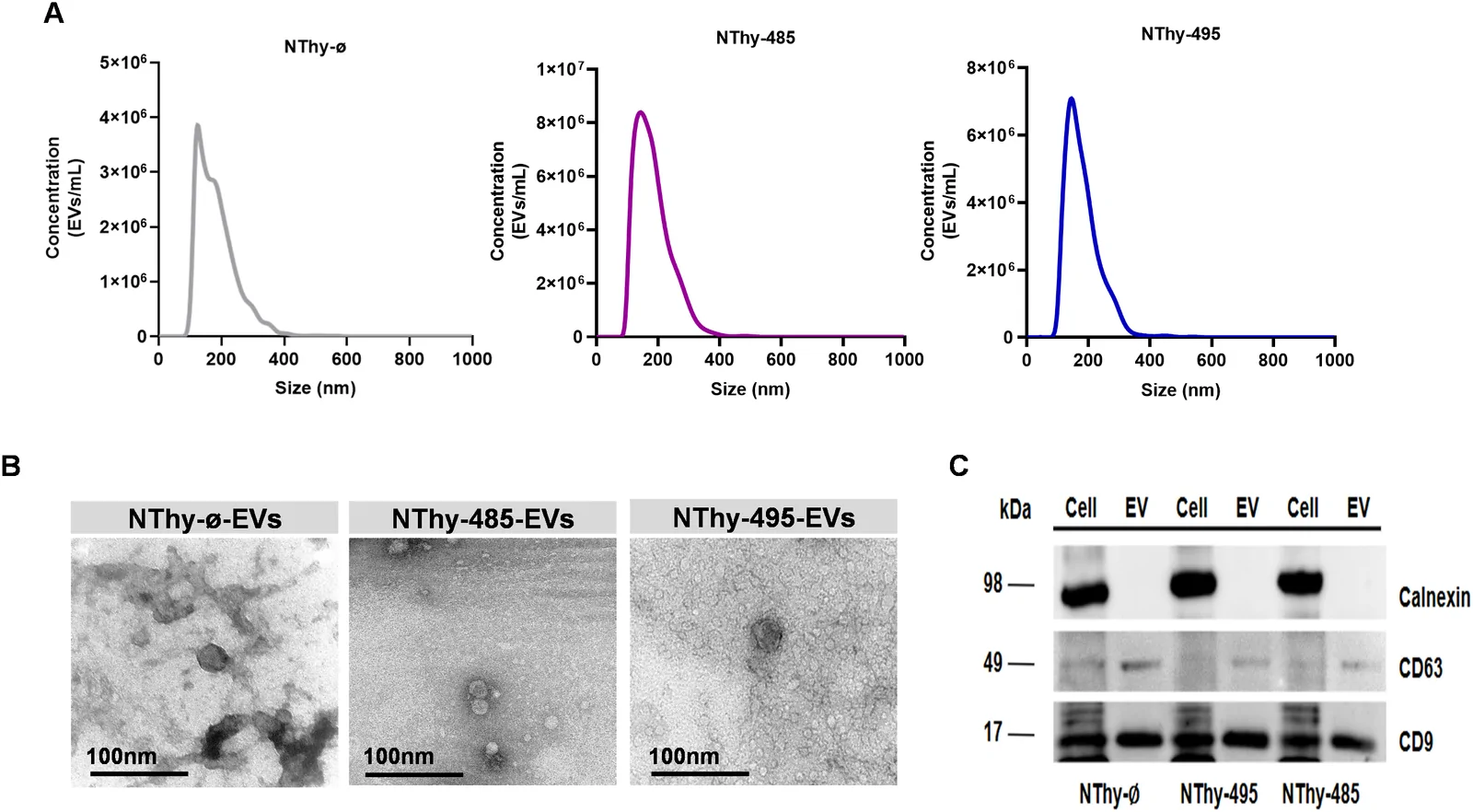
Characterization of EVs released by non-tumoral cells overexpressing *miR-485-5p* and *miR-495-3p*. **(A)** EVs concentration by NTA. **(B)** Transmission Electron Microscopy of isolated EVs. Scale bar: 100 nm. **(C)** Classical EV markers by Western Blot using equal volumes of EV. Data are presented as means ± SD of three independent experiments (n=3).

### 3.3 miRNA-enriched EVs modulate the progression of PTC *in vitro*

Next, to gain insight into whether recipient tumor cells could incorporate miRNAs released by the NThy-ORI cells into the CM, including EVs-encapsulated miRNAs, we evaluated the levels of *miR-485-5p* and *miR-495-3p* in PTC cells following treatment with NThy-ø-CM, NThy-485-CM, and NThy-495-CM. Treatment of TPC-1 and BCPAP tumor cells with NThy-485-CM resulted in a significant increase in intracellular *miR-485-5p* levels, while BCPAP cells treated with NThy-495-CM similarly showed elevated levels of *miR-495-3p* (**Figure 3A**). These findings demonstrated that tumor recipient cells could efficiently uptake the secreted miRNAs released into the extracellular environment, including both EV-encapsulated and extravesicular miRNAs potentially released in the CM.

**Figure 3:**
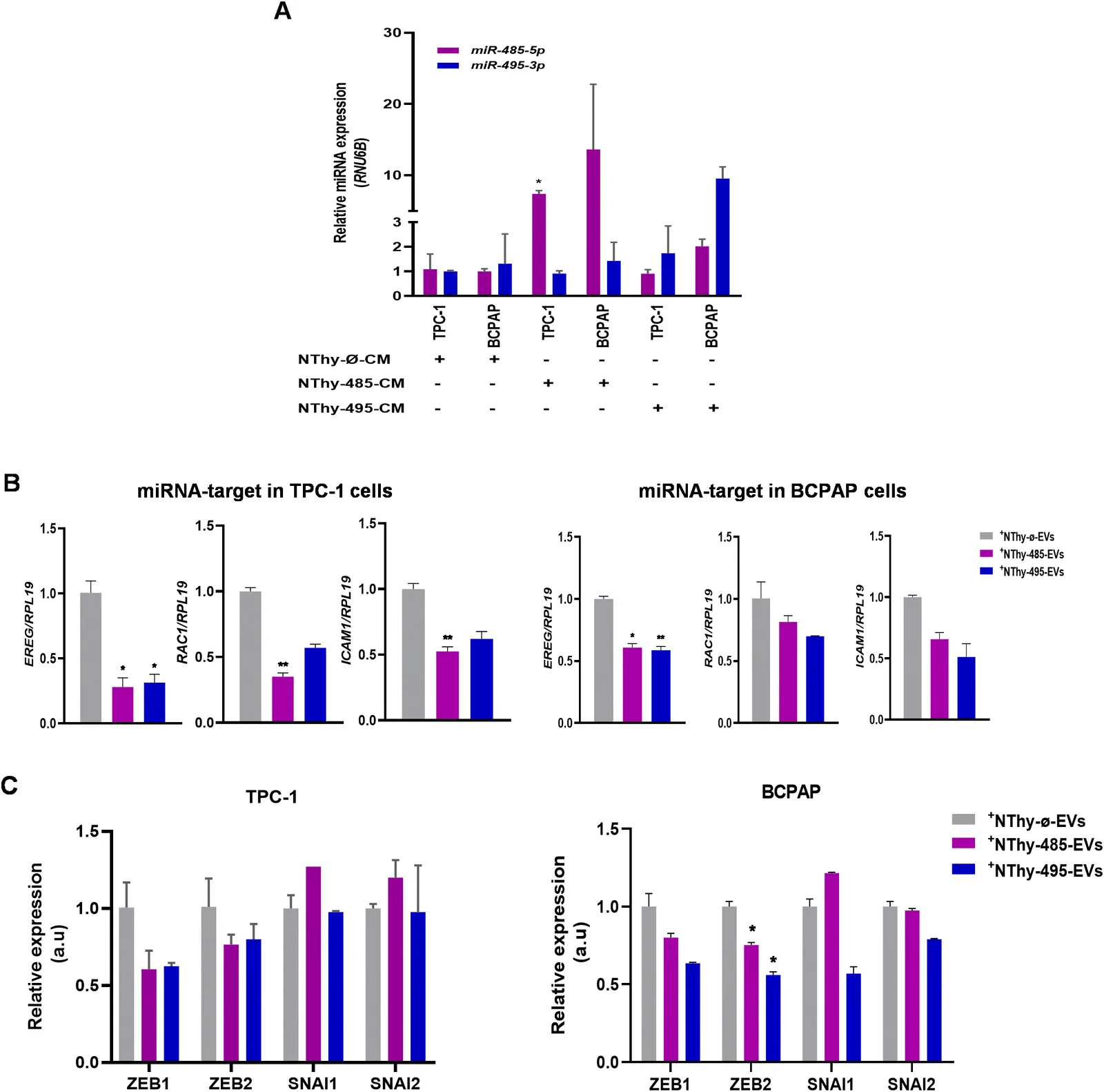
EV-mediated delivery of *miR-485-5p* and *miR-495-3p* reduces EMT-associated gene expression **(A)** *miR-485-5p* and *miR-495-3p* expression in TPC-1 and BCPAP tumor cells after treatment with conditioned medium from NThy-ø, NThy-485, and NThy-495 cells. *RNU6B* was used as an endogenous control. The treated groups were compared to the control group (NThy-ø-CM). **(B)** Relative expression of predicted target genes in tumor cells after treatment with miRNA-enriched EVs. **(C)** Expression of *ZEB1*, *ZEB2*, SNAI1, and *SNAI2* after treatment with miRNA-enriched EVs. Cells treated with EVs from cells transfected with the empty vector (NThy-ø-EVs were used as a control). The *RPL19* gene was used as an endogenous control. Data are presented as means ± SD of technical duplicates from three independent experiments (n=3). Statistical analysis was carried out using two-way ANOVA, with Dunnett’s post-test. *p<0.05.

We then sought to test our hypothesis by further evaluating the ability of miR-485- and miR-495-enriched EVs to post-transcriptionally modulate the expression of known target genes within the recipient cells. As previously demonstrated by our group, *EREG*, *RAC1*, and *ICAM1* are downregulated by *miR-485-5p* or *miR-495-3p* overexpression in thyroid cancer cell lines *in vitro* (19, 20). The treatment with EVs from NThy-485 and NThy-495 cells downregulated these target genes in two different PTC cell lines (**Figure 3B**). Additionally, the treatment with NThy-485- and NThy-495-derived EVs also induced significant changes in the expression of key epithelial-mesenchymal transition (EMT) genes, such as *ZEB1, ZEB2*, *SNAI1*, and *SNAI2* (**Figure 3C**). No significant changes were observed in the expression of EMT genes in TPC-1 cells. These results indicate that *miR-485-5p* and *miR-495-3p* delivered by EVs are internalized and remain biologically active in recipient cells. Importantly, EVs were previously treated with RNase A to remove potential extravesicular miRNAs present in the CM.

Given the post-transcriptional regulation driven by miRNA-enriched EVs, we next sought to investigate whether these EVs could functionally affect their migratory and invasive behavior. Both EV treatments substantially impaired the migratory capacity of TPC-1 and BCPAP tumor cells in scratch, as shown in **Figure 4**. Finally, NThy-485- and NThy-495-EVs significantly reduced migration and invasion in both TPC-1 and BCPAP cells compared to NThy-ø-EVs in the Transwell assay (**Figure 5**). Altogether, our findings indicate that EVs derived from non-tumor NThy-ORI cells efficiently deliver tumor suppressor miRNAs that can modulate the migratory and invasive potential of PTC cells.

**Figure 4:**
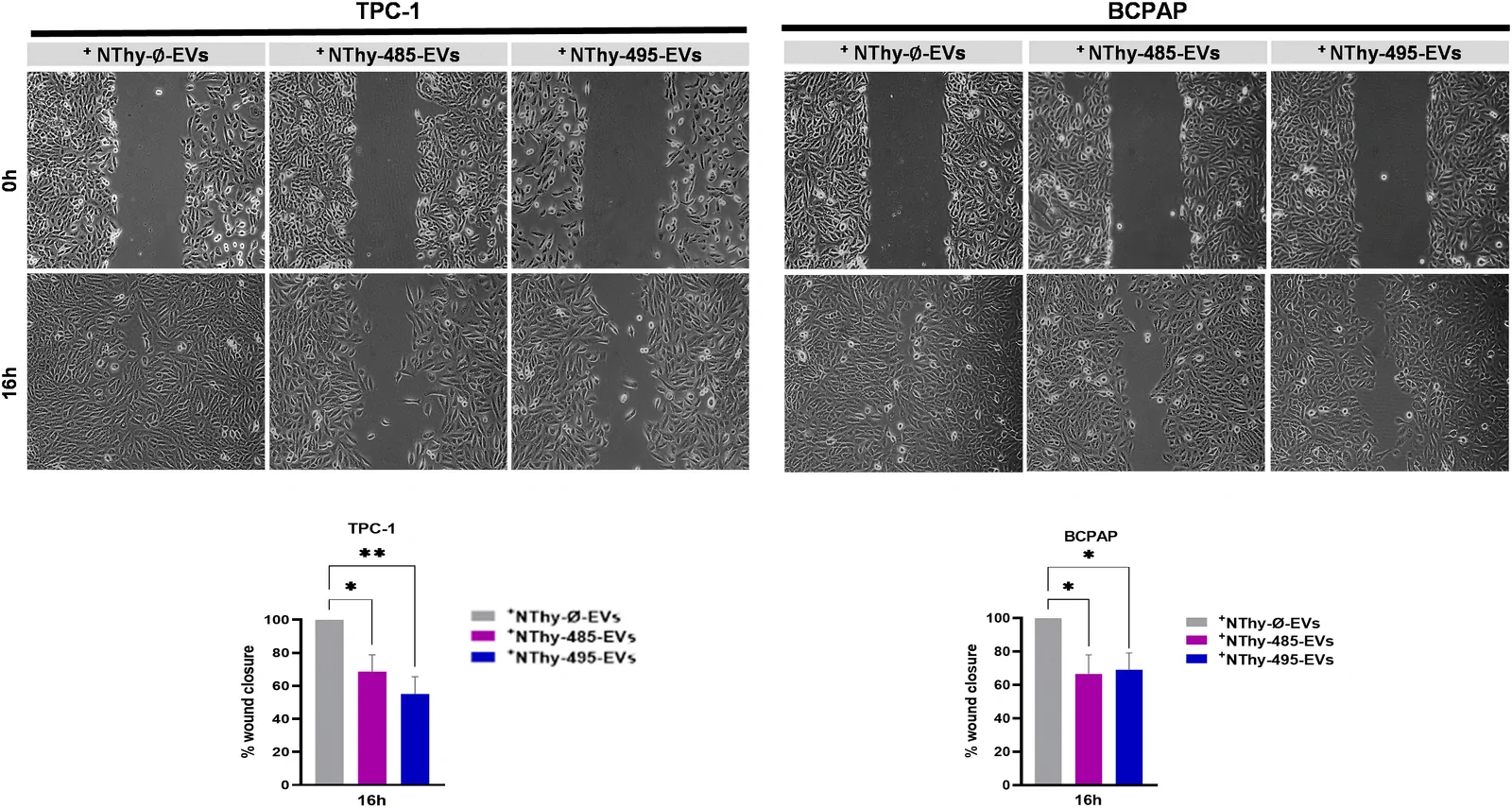
EVs enriched with tumor-suppressor miRNAs impair PTC cells’ migration in the scratch assay. The image shows TPC-1 and BCPAP cells stably treated with EVs after 0 and 16 h of scratch-making (40x magnification). The bar plot below shows the difference in uncovered wounds. Data are presented as means ± SD of technical duplicates from two independent experiments (n=2). Statistical analysis was carried out using two-way ANOVA, with Dunnett’s post-test. In all experiments, the treated groups were compared to the control group (NThy-ø-EVs). *p<0.05; **p<0.001.

**Figure 5:**
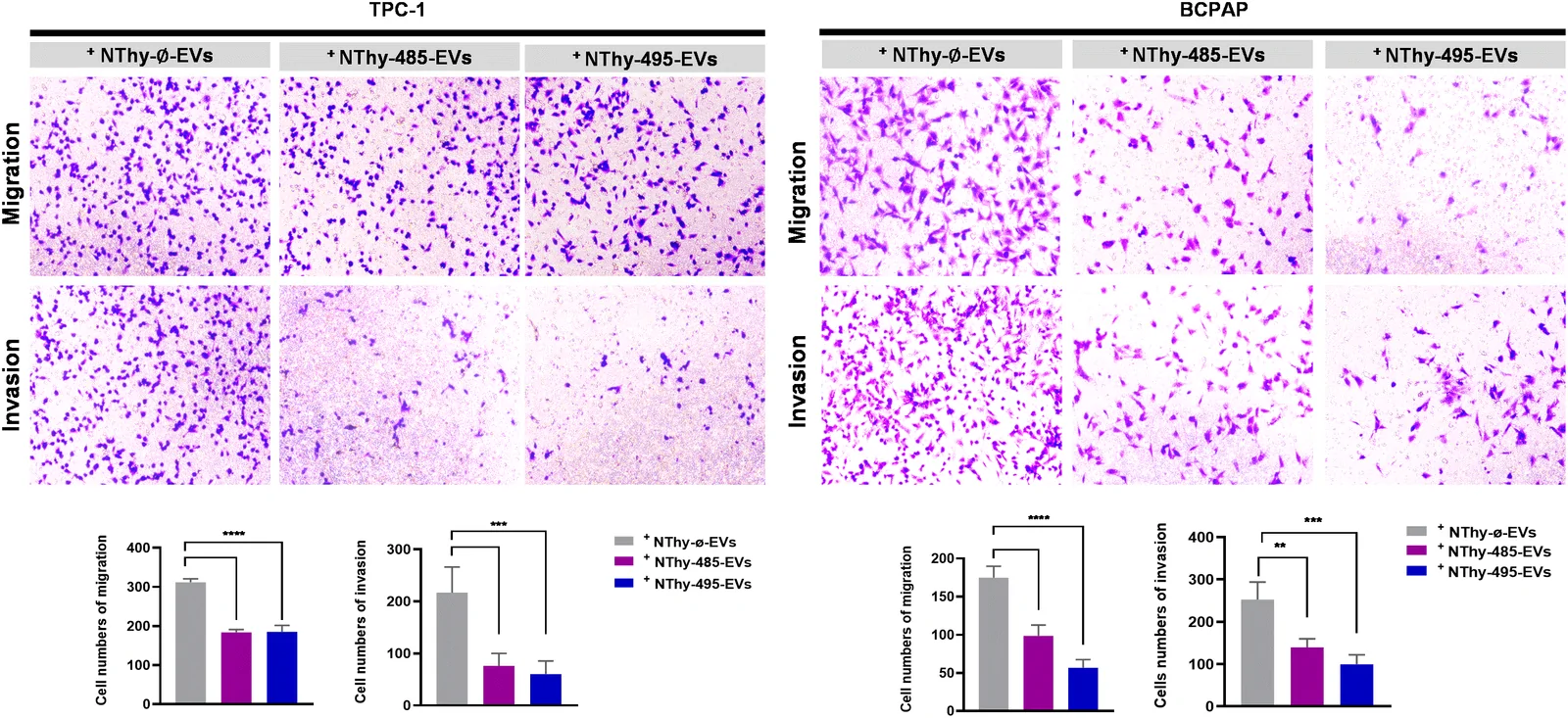
EVs enriched with tumor-suppressor miRNAs impair PTC cells’ migration and invasion. Representative images of TPC-1 and BCPAP cells that migrated/invaded through the Transwell in the migration and invasion assays (100x magnification). Cell numbers obtained after treatment with NThy-ø-EVs, NThy-485-EVs, and NThy-495-EVs. The bar plot below shows the count of cells that migrated/invaded. Data are presented as means ± SD of technical duplicates from two independent experiments (n=2). Statistical analysis was carried out using two-way ANOVA, with Dunnett’s post-test. In all experiments, the treated groups were compared to the control group (NThy-ø-EVs). **p<0.005; ***p<0.001; ****p<0.0001.

## 4 Discussion

The use of EVs as miRNA delivery systems has been widely explored for the treatment of several diseases, including cancer (27–29). However, their potential as vehicles for miRNAs in thyroid carcinoma remains poorly explored. In this work, we demonstrate that non-tumoral thyroid cells overexpressing two different tumor suppressor miRNAs produce and release EVs enriched with these molecules, which can be transferred to recipient cells and modulate key processes involved in PTC carcinogenesis *in vitro*.

Changes in miRNA expression have been associated with the development and progression of different types of cancer (30). As key regulators of gene expression, miRNAs can function as either oncogenes or tumor suppressors, depending on their target genes. Our group has been dedicated to exploring the role of miRNAs from the DLK1-DIO3 region in PTC tumorigenesis and progression. We recently demonstrated that two miRNAs from this region, *miR-485-5p* and *miR-495-3p*, play critical roles in regulating migration, invasion, and epithelial-mesenchymal transition, key processes in PTC progression and aggressiveness (19, 20). These miRNAs have been widely described as tumor suppressors in several types of cancers (31–34).

EV-packaged miRNAs have been suggested as biomarkers and therapeutic tools (7, 13). Once NThy-ORI EVs originate from a non-tumor cell type, we assume that these vesicles would promote a more natural communication with the thyroid tissue, reducing the delivery of foreign miRNAs and growth factors and any eventual oncogenic stimulus or stress response. Non-tumoral thyroid cells naturally express high basal levels of both *miR-485-5p* and *miR-495-3p* in comparison with PTC cell lines (19, 20). Importantly, overexpression of these miRNAs in the normal follicular thyroid cell line led to increased release of these molecules into the CM and EVs. Furthermore, the released miRNAs through EVs are efficiently taken up by recipient tumor cells, supporting the evidence that miRNAs can be transferred to recipient cells.

To confirm whether tumor suppressor miRNAs could be functionally transferred using NThy-ORI-derived vesicles, two PTC cell lines with different genetic backgrounds were used. Our results demonstrate that the EV-packaged *miR-485-5p* or *miR-495-3p* can successfully post-transcriptionally modulate target genes and significantly impair crucial biological processes for tumor progression: cell migration and invasion. *EREG*, *RAC1*, and *ICAM1* were previously reported as targets of *miR-485-5p* and *miR-495-3p* and are crucial for tumor progression in different types of cancer (19, 20, 35–37). EREG is associated with cell proliferation and migration in different types of cancer through activation of pathways such as MAPK and PI3K/AKT (35). *RAC1* and *ICAM1* are also implicated in the modulation of cell migration, invasion, and adhesion (36, 38). The findings align with previous results showing the transfer of extracellular miRNAs to other cells and participation in cellular communication in several biological processes, including differentiation, proliferation, and cell death, and fortify the therapeutic potential of EVs as a natural system for miRNA delivery in the context of PTC (37, 39, 40).

In addition to the modulation of EMT-related genes, particularly in BCPAP cells, multiple molecular mechanisms contribute to the reduced migration and invasion observed in both TPC-1 and BCPAP cell lines *in vitro*. Since the acquisition of migratory and invasive behavior is a key step in tumor progression, these results highlight the potential of miRNA-enriched EVs as modulators of cell behavior. In lung cancer, *miR-485-5p* was described as a modulator of EMT and cell migration by regulating the expression of the target gene IGF2BP2 (41). Similarly, *miR-495-3p* regulates migration and invasion in prostate cancer cells through Akt and mTOR signaling (42). Previously, we have shown that overexpression of *miR-485-5p* impairs proliferation and migration, while *miR-495-3p* inhibits both cell migration and invasion in PTC cells (19, 20). Here, we extend these findings by demonstrating that these effects can be reached through EV-mediated delivery. Importantly, although our main aim was to investigate EVs enriched with tumor-suppressive miRNAs as a delivery system for PTC, we cannot exclude the possibility that the biological effects observed in this work may also result from the combined action of miRNA-enrichment with other alterations in the composition of EVs secreted by donor cells.

As far as we know, we are the first to demonstrate EV-enrichment from thyroid non-tumoral cells without direct transfer methods, such as electroporation or mimetics, supporting a natural and less labor system for miRNA packaging. Altogether, our findings show that a simple strategy was sufficient to produce functionally active, miRNA-enriched EVs capable of modulating tumor cell behavior *in vitro.* It is important to notice that this minimal approach opens the possibility for further optimization, such as double-transfections and multi-step processing to better enrich EVs, which could be explored to improve the efficiency or specificity of EV-mediated delivery for PTC in further *in vitro* and *in vivo* experiments.

Our results suggest the potential of EVs as modulators of the tumor microenvironment. The ability to influence target gene expression, in which *miR-485-5p* and *miR-495-3p* play roles in specific processes, such as migration and invasion, is consistent with our previous findings (19, 20). Nonetheless, limitations persist in our work. For instance, the stability, biodistribution, pharmacokinetics, targeted delivery, and therapeutic efficacy of EVs require further investigation in appropriate *in vivo* models. Additionally, the differential impact observed between tumor cell lines highlights the importance of considering the tumor genetic background when developing future therapeutic strategies. Therefore, our findings provide proof-of-concept that extracellular vesicles can serve as carriers for the delivery of tumor suppressor miRNAs to papillary thyroid carcinoma cells *in vitro*.

## 5 Conclusion

Taken together, our results demonstrate that plasmid-mediated expression of miRNAs in non-tumoral thyroid cells leads to the production of enriched EVs containing tumor-suppressor miRNAs, which can be transferred to recipient cells and impair important processes of thyroid cancer progression, such as migration and invasion *in vitro*. These results provide a foundation for future *in vivo* assays.

## Data Availability

The datasets presented in this study can be found in online repositories. The names of the repository/repositories and accession number(s) can be found below: https://redu.unicamp.br/dataset.xhtml?persistentId=doi:10.25824/redu/00XKF3, https://doi.org/10.25824/redu/00XKF3.
