# Supplementary material for: Modulation of cell migration and invasion of thyroid cancer cells using miRNA-enriched extracellular vesicles
Source: Front Endocrinol (Lausanne). 2026 Sep 15;17:1884738. doi: 10.3389/fendo.2026.1884738 (PMC13619593; doi:10.3389/fendo.2026.1884738)
Supplement: Supplementary file 1 [file SupplementaryFile1.docx]

**Supplementary Material**

| **Antibody** | Dilution (titer) | Brand |
| --- | --- | --- |
| Anti-CD63 | 1:700 | Thermo Fischer Scientific - PA592370 |
| Anti-CD9 | 1:1000 | Santa Cruz - SC13118 |
| Anti-Calnexin | 1:500 | Abcam – ab58504 |
| Anti-rabbit IgG Peroxidase | 1:5000 | Abcam - AB205718 |
| Anti-mouse IgG Peroxidase | 1:2000 | Cell signaling - 7076s |

**Supplementary Table 1.** Antibodies used for WB.

| **Gene** | Forward primer | Reverse primer | Final Concentration (nM) |
| --- | --- | --- | --- |
| EREG | TACTGCAGGTGTGAAGTGGG | GTGGAACCGACGACTGTGAT | 200 |
| *RAC1* | AGAAGCTGACTCCCATCACC | CTTGAGGCCTCGCTGTGT | 200 |
| *ICAM1* | AGCTTCTCCTGCTCTGCAAC | CATTGGAGTCTGCTGGGAAT | 200 |
| *ZEB1* | GGCATACACCTACTCAACTACGG | TGGGCGGTGTAGAATCAGAGTC | 200 |
| *ZEB2* | AATGCACAGAGTGTGGCAAGGC | CTGCTGATGTGCGAACTGTAGC | 200 |
| *SNAI2* | ATCTGCGGCAAGGCGTTTTCCA | GAGCCCTCAGATTTTGACCTGTC | 200 |
| *RPL19* | AACAAGCGGATTCTCATGG | GCGTGCTTCCTTGGTCTTAG | 200 |

**Supplementary Table 2.** Primer sequences used for RT-qPCR.

**Supplementary Figure 1. Tumor cells treated with miR-485-5p- and miR-495-3p-enriched EVs**. TCP-1 and BCPAP cells (10⁴ cells/well) were seeded in technical triplicate in 12-well plates and incubated with NThy-ø-EVs, NThy-485-EVs, and NThy-495-EVs for up to 72 hours, with daily addition of the EVs. After 24, 48, and 72 hours, the cells were harvested and counted using a Neubauer chamber. The graphs represent the cell numbers obtained from the counts. Data are presented as means ± SD of technical triplicates from two independent experiments.
